# Fine Needle Aspiration Cytology of the Breast: The Nonmalignant Categories

**DOI:** 10.4061/2011/547580

**Published:** 2011-05-19

**Authors:** Paulo Mendoza, Maribel Lacambra, Puay-Hoon Tan, Gary M. Tse

**Affiliations:** ^1^Department of Anatomical and Cellular Pathology, Prince of Wales Hospital, The Chinese University of Hong Kong, Shatin, Hong Kong; ^2^Department of Pathology, Singapore General Hospital, Outram Road, Singapore 169608

## Abstract

Currently, accurate diagnosis of breast lesions depends on a triple assessment approach comprising clinical, imaging and pathologic examinations. Fine needle aspiration cytology (FNAC) is widely adopted for the pathologic assessment because of its accurracy and ease of use. While much has been written about the atypical and maliganant categories of FNAC diagnosis, little covers the non-malignanat category which represents a sheer number in all FNAC cases. Moreover, any false-negative diagnosis of the non-malignant cases may lead to missed diagnosis of cancer. This paper aims to discuss the issues of smear adequacy, the cytologic features of benign breast lesions and the dilemma of a false-negative aspirate. Much has been suggested about the smear adequacy criterion, including quantifying epithelial clusters, whereas others advocate basing adequacy on qualitative quantum of using noncellular features of FNAC. Various benign lesions could be easily diagnosed at FNAC; however, they have cytologic features overlapped with malignant lesions. False negativity of FNAC does occur; this could be caused by either “true” false-negative cases attributed to suboptimal sampling technique, poor localization of the mass or nonpalpable lesions or “false” false-negative cases due to interpretational errors. Though false-positive cases are less commonly found, they will also be discussed briefly.

## 1. Introduction

Fine needle aspiration cytology (FNAC) has become popular as a valuable tool in preoperative assessment of breast masses, and it shows high accuracy, sensitivity, and specificity. It has gained popularity due to its fast and easy approach, being inexpensive, and can be performed with little complications. To differentiate benign from malignant lesions is one of the major goals of FNAC. In the evaluation of breast masses, the time honored triple assessment combines clinical, radiological, and pathological information, and FNAC, together with core needle biopsy, is the initial pathological investigative methods of choice. Much confidence has been placed on this approach for it can obviate standard excisional biopsy when all three components of the triple test are conclusively negative or positive [1]. Nevertheless, in FNAC of breast lesions, there are instances where the differentiation of benign and malignant is not possible. This problem arises when paucity of specimen sampling is encountered or there is a morphological overlap between benign and malignant lesions (e.g., atypical hyperplasia and low-grade carcinoma in situ, or in papillary lesions). As a result and to accommodate these problematic areas, cytological reporting categories are used to objectively describe their features in cytological terms and to incorporate the groups with uncertainties. The most commonly used categorization is a five-tier system, with categories ranging from insufficient materials (C1), benign (C2), atypical (C3), suspicious of malignancy (C4), or frankly malignant (C5) (Table 1) [2]. This categorization helps the cytopathologists to define the uncertain areas, and the clinicians to offer further investigation like excisional biopsy judiciously. This categorization was initiated by the national coordinating committee for breast screening and the UK national breast screening program and serves as a common dialect among all breast health care professionals involved in breast management.

**Figure 1:**
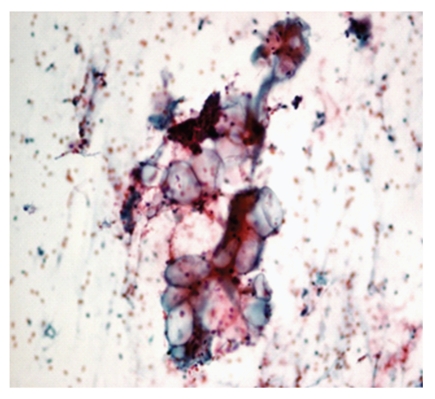
Photomicrograph of hypocellular smear, Pap, 10x. C1, Hypocellular smear.

Under this categorization, C1 is inadequate aspirate smear due to hypocellularity, aspiration, smearing or staining errors. Most often, it is the degree of cellularity of the epithelial cells that is inadequate [2] (Figure 1). The exact definition of what constitutes an inadequate aspirate remains an enigma, and this subjective issue is best determined by the interpreter of the aspirate, whether or not a confident diagnosis could be made basing on the quantity of the materials aspirated. C2 category is for smears that are usually cellular, showing the characteristic patterns of different benign lesions. No atypical or malignant features are present. Usually duct configurations, myoepithelial cells, and bipolar nuclei are visible. Inflammatory background is commonly encountered. In contrast, C3 and C4 are the grey zones. C3 presents the characteristics of a benign smear and yet there are features that are not usually seen in clearly benign specimens such as cellular crowding, pleomorphism, and discohesion. C4 is reserved for aspirate where atypical features are obvious but factors such as poor preservation, hypocellularity, or components of a benign smear are present, thus precluding a firm malignant diagnosis to be made. This ambiguity shows the importance of correlation with other disciplines. It also emphasizes not to stretch the result of FNAC beyond the capabilities and experience of the interpreter to reduce both positive and negative errors [2]. C5 category consists of cellular aspirate with evidently malignant cytologic features. As much has been discussed on the atypical, suspicious, and malignant categories, this paper will be limited to the adequate (or inadequate) and benign categories together with the false negative and false positive cases.

## 2. Adequate FNAC

The adequacy of FNAC is dependent on multiple factors. The rate of inadequate aspiration ranges from 0.7% to 25.3% (Table 2), and this is influenced by the nature of the lesion, the available technology, and the experience and preference of the operator [2]. It was reported that the nature of the lesion was the most common cause of inadequacy of FNAC, accounting for 68% of the inadequate aspirates, followed by the experience of the aspirator that accounted for 32% of the inadequacy rate [3]. During the procedure, patient's cooperation is valuable, and a well-informed patient with good rapport with the operator for FNAC would greatly facilitate the procedure and improve the outcome in terms of adequacy. Thus, each procedure should be patterned and restricted to clinically and radiologically appropriate scenarios [2]. Some studies advocated that both aspirator and interpreter should ideally be the same, as the number of inadequate aspirates was far lower and the accuracy of diagnosis was higher when the same person aspirated and reported on the specimens [2, 4, 5]. The mean frequency of unsatisfactory aspirates by a nonpathologist was twice that when performed by a pathologist [6]. 

Unanimous definition of specimen adequacy in breast FNAC has not been reached so far. The National Cancer Institute (NCI) definition of adequacy was one that led to resolution of a problem presented by a lesion in a particular patient's breast [7]. This definition was somewhat vague, being devoid of a quantifiable clause, but had the advantage of being very flexible and gave the aspirator the full mandate in deciding whether the cytologic features of the aspirate were consistent with the clinical findings and deemed adequate [8]. This would be particularly useful when both the aspirator, and interpreter of the sample were the same. 

Most cytopathologists agree that a number of related parameters are significant determinants of the adequacy of breast FNAC, and these include clinical and imaging findings, size of the lesion, aspiration characteristics, experience of the aspirator, and the number of the needle passes [9]. Nevertheless, many authors considered epithelial cell clusters as the most important adequacy criteria. Studies demonstrated that an appropriate number of epithelial cell clusters could be an important factor in lowering the false-negative diagnosis rate in palpable and nonpalpable breast masses [9–13]. It was further suggested that a cut-off of six epithelial cell clusters may provide a reasonable balance between reduction of false-negative FNAC smears and an increase in the rate of inadequate smears [13]. Since diagnosing malignancy involves evaluation of the cytologic features of the epithelial cells, quantification of epithelial cells in the smears is most likely helpful [9]. Other authors however, proposed not to require a minimum number of ductal epithelial cells as an adequacy criterion, and the assessment relied more on the noncellular features of FNA such as confidence and experience of the clinician or operator with regard to needle placement, resistance of the mass to the needle, and correlation with the clinical and physical findings [8], that is, using a triple assessment approach. Argument for this approach was that if one was to apply a specific number of ductal epithelial cell clusters [3–10], up to 35–40% of the true negative FNAC using a nonquantitative method would become unsatisfactory, forcing patients to undergo more expensive and possibly unnecessary work ups [8]. Typical examples would be in breast cysts, in which the aspirates usually yield histiocytes without epithelial cells. Rendering an inadequate diagnosis in an aspirate that collapsed a cyst yielding significant amount of serous fluid would make correlation with the clinical parameters difficult. Similar situations would be seen in postmastectomy scars with fibrosis, in which the hardened fibrotic area gives very low yield, and labeling such as inadequate may cause anxiety to the patients and prompt unnecessary subsequent investigations or excisions. 

In reality, the issue is not in choosing to which school of thought should one affiliate in defining an adequate smear. A more practical approach is to consider the results of the triple test and the appearance of the epithelial clusters.

## 3. Benign FNAC

**Figure 2:**
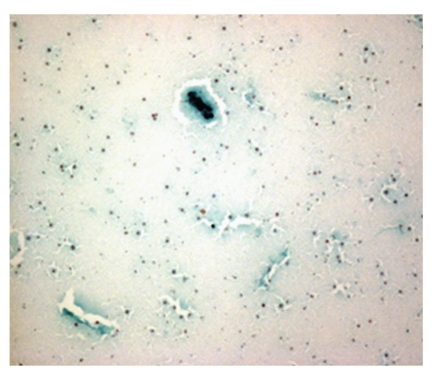
Photomicrograph of cyst contents composed of scattered macrophages and clusters of benign ductal cells, Pap, 4x. Cyst contents: scattered macrophages and few clusters of benign ductal cells.

The bulk of breast FNAC diagnoses are benign, accounting for 24–77.5% of cases (Table 3). Fibrocystic changes present a spectrum of histological features that may sometimes be associated with calcification. Cystic changes represent a common finding. Characteristically, the size of the cyst varies in between consultation visits, giving the clinician further hint on its benign nature, especially when accompanied by imaging studies. In most situations, the aspirated cyst fluid may not be routinely submitted for cytologic evaluation, except when the fluid is blood stained, cloudy, or turbid or when the masses remain uncollapsed and palpable after the aspiration. Most of the time, the smears would only show macrophages mixed with other inflammatory cells, confirming the cystic content nature of the lesion. Ductal epithelial and myoepithelial cells are also commonly seen in cyst aspirate, mostly as small balls and clusters mixed with the macrophages (Figure 2). Apocrine cells lining cyst cavity may exfoliate, showing the characteristic eosinophilic cytoplasm and round nuclei with distinct nucleoli. The above findings of apocrine cells, macrophages, and ductal cells are the characteristic features of a nonproliferative type of fibrocystic changes, which yields only scanty materials. When there is a significant epithelial proliferative component, sheets and tight clusters of cells are usually prominent. The presence of atypia in these cellular clusters may be further evaluated basing on cellular and nuclear spacing, multiple nucleoli, and character of chromatin materials. When these cytologic features are encountered, intraductal papilloma and fibroadenoma are some of the differentials that need to be ruled out. Though cytologically indistinguishable from proliferative fibrocystic changes, intraductal papilloma is often accompanied by clinical history of nipple discharge and a palpable subareolar mass.

Another potential source of confusion rises when there is the presence of proteinaceous fluid in the background, being associated with epithelial cells that are large, with enlarged nuclei, eosinophilic nucleoli, and vacuolated and wispy cytoplasm. The nuclear features may appear worrisome. Nevertheless, one should also be on the outlook for lactational changes, and the appropriate history has to be sought to avoid a false-positive diagnosis [2]. FNA plays a significant role when a discrete nodule appears during pregnancy or lactation. This spares the pregnant patient from the pain and complications of excision. 

**Figure 3:**
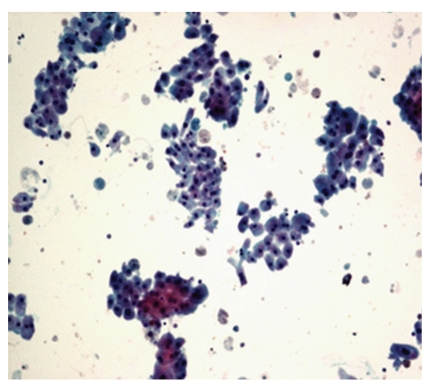
Photomicrograph of apocrine cells with granular cytoplasm and mild anisonucleosis, Pap, 10x. Apocrine cells: granular cytoplasm and mild anisonucleosis.

In more florid examples, thickening of the wall due to papillary apocrine change may cause papillary clusters with the same cytoplasmic and nuclear details to be present [2]. Not all smears from cyst aspirate are easy to evaluate. Apocrine cells, when degenerated will most often appear atypical especially if it has progressed over time to the phase of chromatin clumping with associated anisonucleosis [2], and these could potentially be labeled as suspicious (Figure 3). When there is infection or prior rupture of the cyst, the aspirated fluid may be turbid or milky. Such aspirates often contain degenerated cells and debris in an abundant background of inflammatory cells. In this situation, it needs to be differentiated from the rare squamous carcinoma, which may present with features akin to inflamed cyst. 

Among the solid breast lesions, fibroadenoma is most common especially in women who are less than 40 years old. The clinical presentation is very characteristic, and correct clinical diagnosis can often be made. Radiologically, it is described as a low density mass with well-defined margins. Calcification may not often be present in fibroadenoma especially in the young age group, but among older population a popcorn calcification is characteristic [14]. Multiple fibroadenomas are seen in 15 to 20% of the cases [2]. 

**Figure 4:**
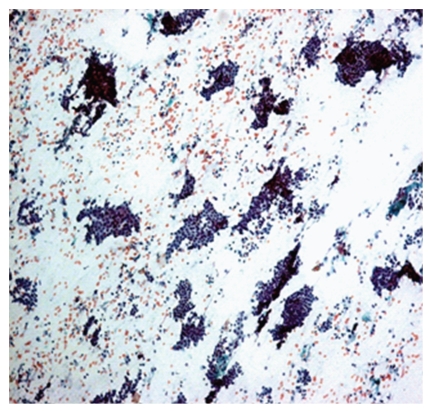
Photomicrograph of hypercellular smear with monolayered sheets of ductal cells in fibroadenoma, Pap, 10x. Fibroadenoma: hypercellular smear with monolayered sheets of ductal cells.

**Figure 5:**
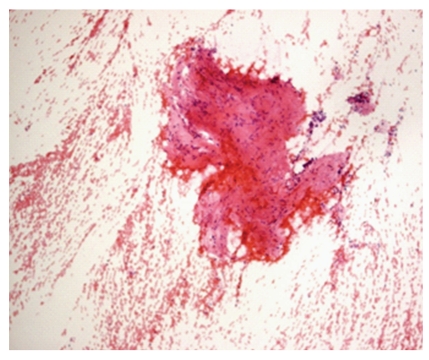
Photomicrograph of stromal fragments in fibroadenoma, H&E, 10x. Fibroadenoma: stromal fragments.

**Figure 6:**
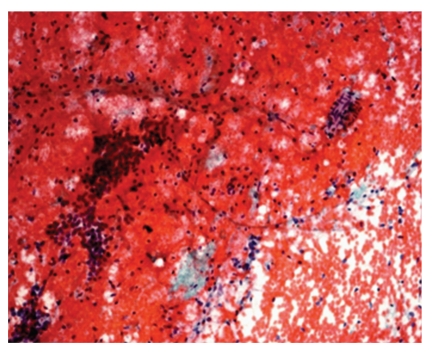
Photomicrograph of numerous bipolar cells in fibroadenoma, H&E, 10x. Fibroadenoma: bipolar cells in the background.

**Figure 7:**
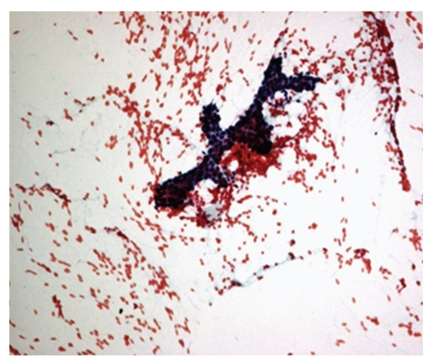
Photomicrograph of antler-horn configuration of ductal cells in fibroadenoma, Pap, 10x. Fibroadenoma: antler-horn configuration.

**Figure 8:**
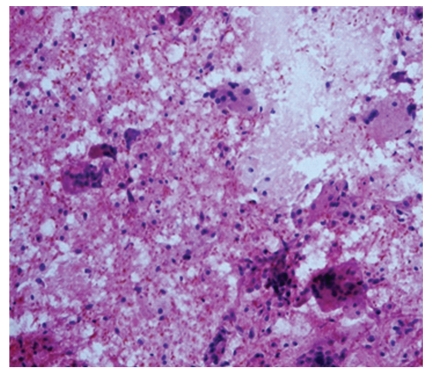
Photomicrograph of giant cells in fibroadenoma, H&E, 20x. Fibroadenoma: stromal giant cells.

FNAC diagnosis of fibroadenoma is highly accurate. Lopez-Ferrer reported a 79.3% predictive value out of 362 fibroadenoma aspirates with most diagnostic errors occurring in the older age group [15]. Cytologically, aspirates are hypercellular with characteristic monolayer sheets of benign-looking epithelial cells mixed with myoepithelial cells. These sheets are often described as “staghorn”, having antler-like configuration on its edges (Figure 4). This pattern reflects the configuration of ducts as observed on histological sections [2, 16]. Cellular cohesiveness is often appreciated in the aspirate smear. Accompanying the epithelial cells are the fibrillar stromal materials which may vary in cellularity and sometimes show myxoid change (Figure 5). Commonly, the background of the aspirate is composed of numerous naked/bipolar nuclei (Figure 6). This is one of the characteristic cytologic features of fibroadenoma. The added presence of large number of bipolar nuclei in the background of smear is a reliable feature in favor of fibroadenoma [2]. There are aspirates which may show less pronounced antler horns but this may represent sample from fibroadenoma with pericanalicular pattern. Branching of epithelial sheets is more common if the aspirated sample is from an intracanalicular form (Figure 7). The commonly encountered cytological features of fibroadenoma are fibromyxoid stroma, staghorn clusters, and numerous single bare nuclei, being seen in 92.7%, 73.6%, and 73.6% of cases, respectively [17]. These findings constitute the diagnostic triad for fibroadenoma. There are instances wherein the diagnosis of fibroadenoma on cytology is not straight forward. The absence of any components of the diagnostic triad and low cellularity are the common causes of pitfalls in missed cytodiagnosis of fibroadenoma [17] (Table 3). Giant cells are uncommonly seen in fibroadenomas (Figure 8). In the report of Kollur and El Hag, it showed an increased incidence, being present in 31.8% of the aspirated cases [17]. These giant cells are variable in appearance, were thought to be stromal in origin [18, 19], and are of little prognostic significance. Most series reported the presence of these stromal giant cells being present in fibroepithelial lesions of the breast, but were more common in phyllodes tumor than fibroadenomas [18, 19]. Sometimes, giant cells may indicate an extra-tumoral reactive process in the surrounding breast tissue which may be due to palpation granuloma or fat necrosis [17]. It is a known fact that fibroadenoma is difficult to distinguish from phyllodes tumor using aspiration cytology but there are some features that are more characteristic to phyllodes tumors that will support its diagnosis on cytology. A cellular aspirate with numerous plump and spindly nuclei, pronounced of hypercellularity of stromal fragments, and presence of atypia are the key points that support a diagnosis of phyllodes tumor over fibroadenomas. However, these differentiating features may not be present in all cases. The presence of more stromal fragments over epithelial fragments (higher stromal epithelial ratio) and the presence of single columnar cells in the background are some of the “soft signs” reported for the identification of phyllodes tumor over fibroadenoma. In the extremely rare instance in which a malignant phyllodes tumor is encountered, the sarcomatous spindle cells within cellular stromal fragments may be definitive for the establishment of the diagnosis. Fibroadenomas also need to be differentiated from papillomas, by virtue of the fact that the latter show presence of small cell balls or clusters, with either staghorn or papillary configurations in the smears. 

On the whole, FNAC showed a high sensitivity of up to 68–97% in fibroadenomas [15, 17], and it has been demonstrated that the overall cellularity, amount of bipolar nuclei, amount and architectural of epithelium, apocrine metaplasia, nuclear overlapping and pleomorphism, foam cells, and stroma are significant cytologic parameters in distinguishing fibroadenomas from papilloma, fat necrosis, fibrocystic changes, and duct ectasia [16].

**Figure 9:**
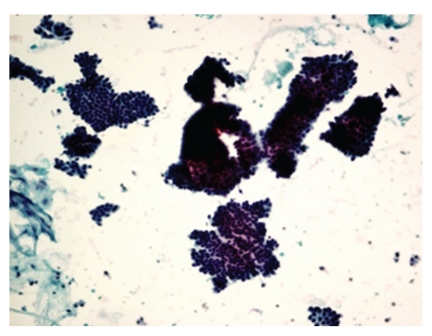
Photomicrograph of papillary fronds in papilloma, Pap, 10x. Papilloma: papillary fronds.

**Figure 10:**
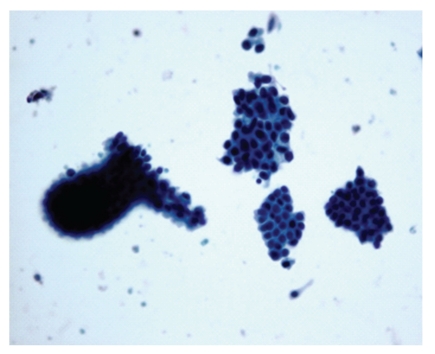
Photomicrograph of cell balls with cytologic atypia in papilloma, Pap, 20x. Papilloma: cell balls with cytologic atypia.

Nipple discharge is one of the alarming complaints that would prompt patients to seek clinical consults. This represents, commonly, a papillary lesion involving one of the major lactiferous ducts [2]. Intraductal papillomas are usually solitary and most often found in the subareolar region. It is relatively common, accounting for 2.5% of all benign breast excisions [16]. It is seen as a well-defined mass which radiologically presents as low-density soft tissue mass with no surrounding architectural distortion or tissue response. Calcification, when present, is usually of the dystrophic and curvilinear type [2]. In addition, papillary fronds reminiscent of staghorn clusters can also be seen in papillomas. It was found that foam cells in association with these fronds is one of the more specific features of differentiating papilloma from fibroadenoma [2, 16]. Papillomas in FNAC may cause diagnostic problems. The accuracy of FNAC in diagnosing papillary lesions and differentiating benign and malignant papillary lesions is low [20]. Among the aspirates diagnosed as atypical, intraductal papilloma represents about 6% [21]. For papillomas, the typical FNAC picture of papillary fronds, cell balls, and columnar cells may not all be seen in the aspirate, which may also be complicated by a hemorrhagic background (Figures 9 and 10). At such the cytologic picture may raise the possibility of a malignancy. Problems also occur when the papillomas are complicated by epithelial hyperplasia, hyalinization, or apocrine changes as these may yield hypocellular to hypercellular smears with pleomorphic cells showing prominent and background necrotic debris [22, 23]. To date, there have been no well-defined cytological criteria to differentiate between benign and malignant papillary lesions. Their significant overlap in terms of architecture and cytological atypia is the primary reasons for not differentiating them cytologically [20, 21].

## 4. False-Negative FNAC

FNAC has irrefutably and significantly contributed to the reduction of excisional biopsies in the assessment of breast lesions, especially in the context of triple assessment [24]. Nevertheless, there still exists a significant false negative rate for FNAC, in the range of 1.2–10.6% (Table 4). These may lead to missed/delayed diagnosis and treatment [25], sometimes with adverse clinical outcome. This has become a major concern, prompting, on the side of the laboratory and pathologist, a re-evaluation of the adequacy limitation, referral system, and processing techniques. Previous studies have demonstrated that the sensitivity, specificity, and accuracy of breast FNAC all ranged from 77% to 100% [24, 26–29]. 

The underlying causes for false negativity can be grouped into diagnostic errors and true false negative factors. Diagnostic errors can be attributed to lack of training, overload of cases, and miscorrelation with the patient's clinical and radiologic findings [6]. In the true false negative factors, the denominators are poor sampling technique, mislocalization of the tumor, or the presence of a well-defined tumor demonstrating minimal atypia [29, 30]. The widespread adoption of breast screening and advances in imaging techniques also resulted in the detection of small lesions, and understandably, FNAC of these small lesions has a significant risk of missing these lesions, leading to potentially false-negative results.

Nonpalpable lesions constitute a specific category of screen detected lesion. In one study, 21% of false negative breast FNAC was due to nonpalpable tumors [31]. The main problem associated with FNAC of nonpalpable lesions was the variable but sometimes unacceptably high rate [32] of inadequacy. An inadequacy rate as high as 34–58% had previously been reported [33, 34], and the lowest reported inadequacy rate was around 10% [35, 36]. Attaining adequacy in the aspirates of these nonpalpable lesions poses greater challenge because of their small sizes in many cases, as well as the presence of fibrotic component. Nowadays, management of these lesions always involves CT or ultrasound guidance to better define and localize the lesion upon aspiration. Apart from tumor size, tumor grade was also an important risk factor for false-negative FNAC (Table 5). In Bulgaresi's report of false-negative FNAC reporting, 24.3% were those from special types of tumor, 39% of which were low grade tumors [31]. The resemblance of lobular carcinoma to lymphocytes and its subtle cytologic atypia are well-known diagnostic problems. Ductal carcinoma, not otherwise specified (NOS) subtype, accounted for 2/3 of the cases false-negative cases in another series [37]. As a result, nonpalpable lesions constitute a specific “blind area” not amenable to FNAC, indeed most authors would recommend core needle biopsy for the work up of such lesions [32]. 

## 5. False-Positive FNAC

False-positive diagnosis in aspiration cytology is significantly lower in incidence compared to false-negative cases. From the previous reports, false-positive cases range from 0% to 2% (Table 6), in most studies reporting a 100% positive predictive value. Among the reported cases, the common lesion giving a false-positive aspirate is ductal hyperplasia or lobular hyperplasia. This finding is also in consonance with previous reports that fibrocystic changes and pregnancy-related breast masses account for false-positive findings [25]. In most of the accounted cases, radiologic findings are mostly indeterminate for breast cancer, requiring confirmation by histology.

## 6. Summary

FNAC is an essential component in the preoperative management of breast lesions. Its accuracy, ease of use, and affordability are factors that cause its popularity. The advent of imaging technology together with the clinical expertise of the clinician contributed to its increased sensitivity. The adequacy of smears is influenced by the nature of the lesion, experience of the aspirator, and access to the available imaging modality. An adequate smear can be defined by either quantitative or qualitative means, with advocates for either approach. Nevertheless, the operators' experience and confidence in correlating with the clinical and radiologic findings, the cellularity of smears, and the aspiration technique are always helpful. Exceptions occur in cystic and fibrotic lesions that are inevitably hypocellular. Degenerative changes would render the smear to be difficult to interpret. Benign breast lesions are usually easy to diagnose when their characteristic cytologic patterns are obvious. Hypocellularity, degenerated apocrine cells, necrosis, and epithelial hyperplasia are some of the factors that may be encountered in evaluating a difficult smear, mimicking atypical or malignant lesions. The false-negative cases in breast FNAC, although few, are commonly due to poor sampling technique, poor tumor localization, and the presence of a well-differentiated histology of the tumor. Small tumor size and nonpalpable breast lesions are also commonly associated with false-negative and aspirate inadequacy. Thus in the interpretation of breast FNAC, all these factors should be considered before a benign diagnosis is being rendered.

## Figures and Tables

**Table 1:** Cytology reporting categories*.

C1 Inadequate
C2 Benign
C3 Atypia probably benign
C4 Suspicious of malignancy
C5 Malignant

*From Diagnostic Cytopathology of the Breast by Zakhour and Wells [2].

**Table 2:** Inadequate FNA.

Authors	Inadequate cases (%)	Total number of cases
O'Neil et al. [24]	0.7%	697
Nguansangjam et al. [38]	4.2%	190
Rosa et al. [39]	8%	1583
Day et al. [40]	9%	831
Feichter et al. [41]	16.2%	1003
Zarbo et al. [6]	17%	13066
Park and Ham [37]	25.3%	699

**Table 3:** Benign FNA.

Authors	Benign cases (%)	Total number of cases
O'Neil et al. [24]	24%	697
Ishikawa et al. [25]	47.6%	382
Rosa et al. [39]	60%	1583
Feichter et al. [41]	68.1%	1003
Day et al. [40]	77.5%	831

**Table 4:** False-negative FNA.

Authors	False negative cases (%)	Total number of cases
Rosa et al. [39]	1.2%	1583
O'Malley et al. [12]	1.6%	1005
O'Neil et al. [24]	1.9%	697
Ishikawa et al. [25]	2.2%	382
Arisio et al. [29]	3.9%	1601
Day et al. [40]	5.4%	831
Feichter et al. [41]	9%	1003
Park and Ham [37]	10%	699

**Table 5:** Surgical followup in false-negative cases*.

Follow up tissue diagnosis	Number/percentage of cases
Atypical ductal hyperplasia	1 (5%)
Ductal carcinoma in situ	3 (16%)
Cribriform carcinoma	1 (5%)
Metaplastic carcinoma	1 (5%)
Infiltrating lobular carcinoma	6 (32%)
Infiltrating ductal carcinoma	7 (37%)

Total cases	19

*Rosa [39]. The value of fine needle aspiration biopsy in the diagnosis and prognostic assessment of palpable breast lesions.

**Table 6:** False-positive FNA.

Authors	False negative cases (%)	Total number of cases
Rosa et al. [39]	0%	1583
Day et al. [40]	0%	831
Arisio et al. [29]	0.3%	1601
Feichter et al. [41]	0.5%	1003
Park and Ham [37]	1%	699
Ishikawa et al. [25]	2%	382
